# Effects of preoperative surgeon warm-up in video-assisted thoracoscopic surgery lobectomy

**DOI:** 10.1186/s12893-023-02300-3

**Published:** 2024-01-03

**Authors:** Enjie Wang, Jun Li, Tao Hong, Zexin Xie, Yong Ge, Xiaotong Zhou, Hao Zhang

**Affiliations:** 1 Thoracic Surgery Laboratory, Xuzhou Medical University, Xuzhou, China; 2 Department of Thoracic Surgery, Affiliated Hospital of Xuzhou Medical University, 99 West Huaihai Road, Xuzhou, Jiangsu 221003 China; 3 Department of Thoracic Surgery, Dazhou Third People’s Hospital, Dazhou, China

**Keywords:** Surgical warm-up, Perioperative outcomes, Lobectomy, Lung cancer

## Abstract

**Background:**

In various surgical specialties, preoperative surgical warm-up has been demonstrated to affect a surgeon’s performance and the perioperative outcomes for patients. However, the influence of warm-up activities on video-assisted thoracoscopic surgery lobectomy (VATSL) remains largely unexplored. This study aims to investigate the potential effects of preoperative surgical warm-up on VATSL.

**Methods:**

A cohort of 364 patients diagnosed with lung cancer through pathology and undergoing VATSL at the Thoracic Surgery Department of Xuzhou Medical University from January 2018 to September 2022 were included. Patients were categorized into two groups: the warm-up group, comprising 172 patients undergoing their first VATSL of the day, and the warm-up effect group, consisting of 192 patients undergoing their second VATSL on the same day. Propensity score matching was employed to compare operation times and postoperative complications between the two groups, resulting in 159 matched cases in each group.

**Results:**

There were no statistically significant differences in operation time (154.5 ± 54.9 vs. 147.2 ± 54.4 min, *p* = 0.239) and postoperative complications (including pulmonary infection, atelectasis, long-term pulmonary air leakage requiring incision suture in the operating room, and postoperative pleural effusion) (14:22 cases, *p* = 0.157) between the warm-up and warm-up effect groups.

**Conclusion:**

The findings suggest that preoperative surgical warm-up does not significantly affect the perioperative outcomes of VATSL.

## Introduction

The concept of warming up has been commonly practiced and studied in activities requiring complex psychomotor skills, including sports and musical instrument performance [1]. Warming up has been reported to improve performance, reduce task completion times, alleviate anxiety, and improve the focus of participants [2]. Its application in high-stake, high-performance professions with substantial psychomotor skills proficiency, such as dancers, musicians, sculptors, and painters, has historical roots. [3]. A common thread among all activities that promote use of warm-up includes strenuous physical activity; strenuous mental activity with requirements of cognitive arousal; and ability to perform both within required coordination and task performance constraints.

In complex procedures such as video-assisted thoracoscopic surgery lobectomy (VATSL), surgeons face the challenge of maintaining a high concentration level and possessing expert surgical skills due to the inherent complexity and significant risks involved. There is a prevailing perception, held by both patients and surgeons, that surgeons perform at their peak early in the day, delivering optimal surgical quality and achieving the best postoperative results [4]. Consequently, patients often express a preference for having their surgeries scheduled early in the day. However, the medical and health sectors in China confront issues of limited medical resources and their uneven distribution [5], leading to a concentration of patients in large medical institutions or public hospitals and placing a considerable workload on healthcare professionals [6].

As we know, the roles of surgeons extend beyond surgery to include ward management, student teaching, scientific research, administrative management, and more. The role of preoperative warm-up is to assist the surgeon in completing the transition from their other working states to the focused surgical state. However, whether this transition process impacts short-term patient outcomes remains unclear[7, 8]. Our research aims to elucidate how a preoperative warm-up activity within the surgical case warm-up routine influences the senior-level performance of a thoracic surgeon during VATSL.

## Methods

### Data sources

We retrospectively collected clinical data from 364 patients who underwent VATSL by the same physician at the Affiliated Hospital of Xuzhou Medical University between January 2018 and September 2022. The patients were categorized into warm-up and warm-up effect groups according to the order of surgery on that day. The warm-up group included patients who underwent the first-session VATSL, whereas the warm-up effect group comprised those who underwent the second-session VATSL. The operation time was defined as the interval between skin incision and complete skin suturing. All procedures were conducted by the same thoracic surgeon through video-assisted thoracoscopy (VATS), involving one or two incisions. The warm-up and warm-up effect groups were subsequently matched for propensity scores in age, sex, body mass index (BMI), the American Society of Anesthesiology (ASA) score, age-corrected Charlson comorbidity index (ACCI), number of lymph node dissections, hypertension, diabetes mellitus, coronary artery disease, chronic obstructive pulmonary disease (COPD), surgical anatomic site, and the Eastern Cooperative Oncology Group (ECOG) score. Potential COPD-related confounding effects were eliminated after matching, resulting in two matched groups (n = 159 in each group). Finally, we compared the perioperative outcomes between the two groups.

### Inclusion criteria

Inclusion criteria: (1) patients who received the first- and second-session VATSL of the day; (2) patients aged ≥ 18 years and < 80 years; and (3) patients with a single tumor pathologically confirmed as lung cancer.

### Exclusion criteria

Exclusion criteria: (1) patients with other concurrent tumors or pathologically confirmed benign tumors; (2) patients undergoing two or more simultaneous procedures (wedge resection, segmental resection, and lobectomy); (3) patients undergoing palliative surgery because of poor underlying conditions combined with organ insufficiency or other complications; and (4) patients with conditions such as total thoracic adhesions or doornail lymph nodes that require prolonged surgery.

### Surgical methods

Patients were ventilated in the lateral position under general anesthesia with single lung intubation. The same surgeon conducted single lobectomy and mediastinal lymph node dissection via one or two incisions under VATS. A chest drainage tube (Fr24) was postoperatively placed through the incision. The need for intensive care unit (ICU) admission was evaluated based on intraoperative and postoperative vital signs by an anesthesiologist. Standard postoperative care included routine intravenous drips, additional intramuscular pain medications as needed, early mobilization, chest expansion exercises (coughing), nutritional support, and short-term postoperative antibiotics to prevent infection. The postoperative chest drainage tube was extubated after the extubating criteria were met (no gas leakage from the chest tube at 24 h and no more than 200 ml of drainage fluid [9], with good pulmonary re-expansion on chest X-ray).

### Primary and secondary outcomes

Primary outcomes encompassed operation time and postoperative complications, which included pulmonary infection, pulmonary atelectasis, long-term postoperative pulmonary air leak (defined as persistent postoperative pulmonary air leak for more than 7 days) [10, 11], re-suturing of the incision in the operating room, and postoperative pleural effusion [12]. The time to extubating of the chest drainage tube, postoperative hospital stays, and postoperative ICU admission were considered secondary outcomes.

### Statistical analyses

SAS 9.4 (version 9.4 for Windows, SAS Institute, Inc., Cary, NC, USA) was used for data management and statistical analyses.

Univariate analyses were performed before and after matching. Data conforming to normal distribution were expressed using mean (SD) and a t-test was used for between-group comparisons. Quantitative data that was not normally distributed were expressed using M (P25, P75) and subjected to a rank-sum test for between-group comparisons.

The two groups were treated using propensity score matching (PSM) with a caliper value of 0.25 to balance potential confounding bias from differences in baseline characteristics between the two groups. Subsequently, PSM was applied to baseline information of patients for 1:1 PSM (age, sex, BMI, ASA score, ACCI, number of lymph node dissections, hypertension, diabetes mellitus, coronary heart disease, COPD, surgical anatomical site, and ECOG score) [12–15]. Qualitative data were expressed using n (%) and subjected to the Chi-squared test and Fisher’s exact probability test for between-group comparisons. The test level α was set at 0.05 if not otherwise specified. A *p*-value < 0.05 indicated statistical significance.

## Results

### Baseline data of the patients

We included 364 patients who had undergone VATSL from January 2018 to September 2022 (106 in the right upper lobe; 83 in the left upper lobe; 32 in the right middle lobe; 73 in the right lower lobe; and 70 in the left lower lobe) in this study. The baseline data of the patients are presented in Table 1.

**Table 1:** Baseline characteristics of study subjects [n (%)]

Variables (n = 364)	Subgroups	Statistics [n (%)]
Age (years)		60.5 ± 9.8
Sex	Female	189 (51.9)
	Male	175 (48.1)
BMI		22.3 ± 1.6
ECOG score		0.7 ± 0.7
ASA score		1.3 ± 0.5
ACCI		1.9 ± 1.2
COPD		9 (2.5)
Hypertension		50 (13.7)
Diabetes		27 (7.4)
Coronary heart disease		11 (3.0)
Smoking		82 (22.5)
Alcohol consumption		61 (16.8)
Number of lymph node dissections		5.2 ± 1.3
Surgical sites	Right upper lobe	106 (29.1)
	Left upper lobe	83 (22.8)
	Right lower lobe	73 (20.1)
	Left lower lobe	70 (19.2)
	Right middle lobe	32 (8.8)
Pathology	Adenocarcinoma	340 (93.4)
	Squamous carcinoma	19 (5.2)
	Small cell carcinoma	2 (0.5)
	Neuroendocrine carcinoma	2 (0.6)

Results are expressed as propensity scores and *p-values*, where exceptions are described separately

ASA: American Society of Anesthesiology score; BMI: body mass index; CAD: coronary artery disease; ACCI: age-adjusted Charlson Comorbidity Index; COPD: chronic obstructive pulmonary disease; and ECOG score: Eastern Cooperative Oncology Group score

### Analysis of surgical warm-up

Patients were grouped based on the presence of warm-ups. The first- and second-session groups contained 192 and 172 patients, respectively. Table 2 displays the mismatch comparison of baseline characteristics between the two groups.

**Table 2:** Comparison of the pre-matching characteristics between the two groups [n (%)]

Variables	Warm-up group (n = 172)	Warm-up effect group (n = 192)	*χ* ^*2*^ */t*	*p-values*
**Sex**				
Female	96 (55.81)	93 (48.44)	1.98	0.160
Male	76 (44.19)	99 (51.56)		
**Age (years)**	60.1 ± 10.1	60.8 ± 9.6	0.74	0.463
**BMI**	22.3 ± 1.6	22.3 ± 1.7	0.08	0.934
**ACCI**	1.8 ± 1.1	2.0 ± 1.2	1.21	0.225
**Number of lymph node dissections**	5.2 ± 1.4	5.1 ± 1.3	0.45	0.650
**Hypertension**	23 (13.37)	27 (14.06)	0.04	0.848
**Diabetes mellitus**	9 (5.23)	18 (9.38)	2.27	0.132
**Coronary heart disease**	3 (1.74)	8 (4.17)	1.82	0.178
**COPD**	1 (0.58)	8 (4.17)	—	0.039*
**Surgical anatomic site**			2.13	0.712
Right upper lobe	53 (30.81)	53 (27.60)		
Left upper lobe	36 (20.93)	47 (24.48)		
Right lower lobe	38 (22.09)	35 (18.23)		
Left lower lobe	32 (18.60)	38 (19.79)		
Right middle lobe	13 (7.56)	19 (9.90)		
**ECOG score**	1.0 (0.0, 1.0)	1.0 (0.0, 1.0)	1.41	0.158^#^
**ASA score**	1.3 ± 0.5	1.3 ± 0.6	0.27	0.785

Results are expressed as propensity scores and *p-values*, where exceptions are stated separately. *p-values* > 0.05 indicated minor differences

ASA: American Society of Anesthesiology score; BMI: body mass index; CAD: coronary artery disease; ACCI: age-adjusted Charlson Comorbidity Index; COPD: chronic obstructive pulmonary disease; ECOG score: Eastern Cooperative Oncology Group score

*Note*:*Fisher’s exact probability test; # Rank-sum test

**Fig. 1:**
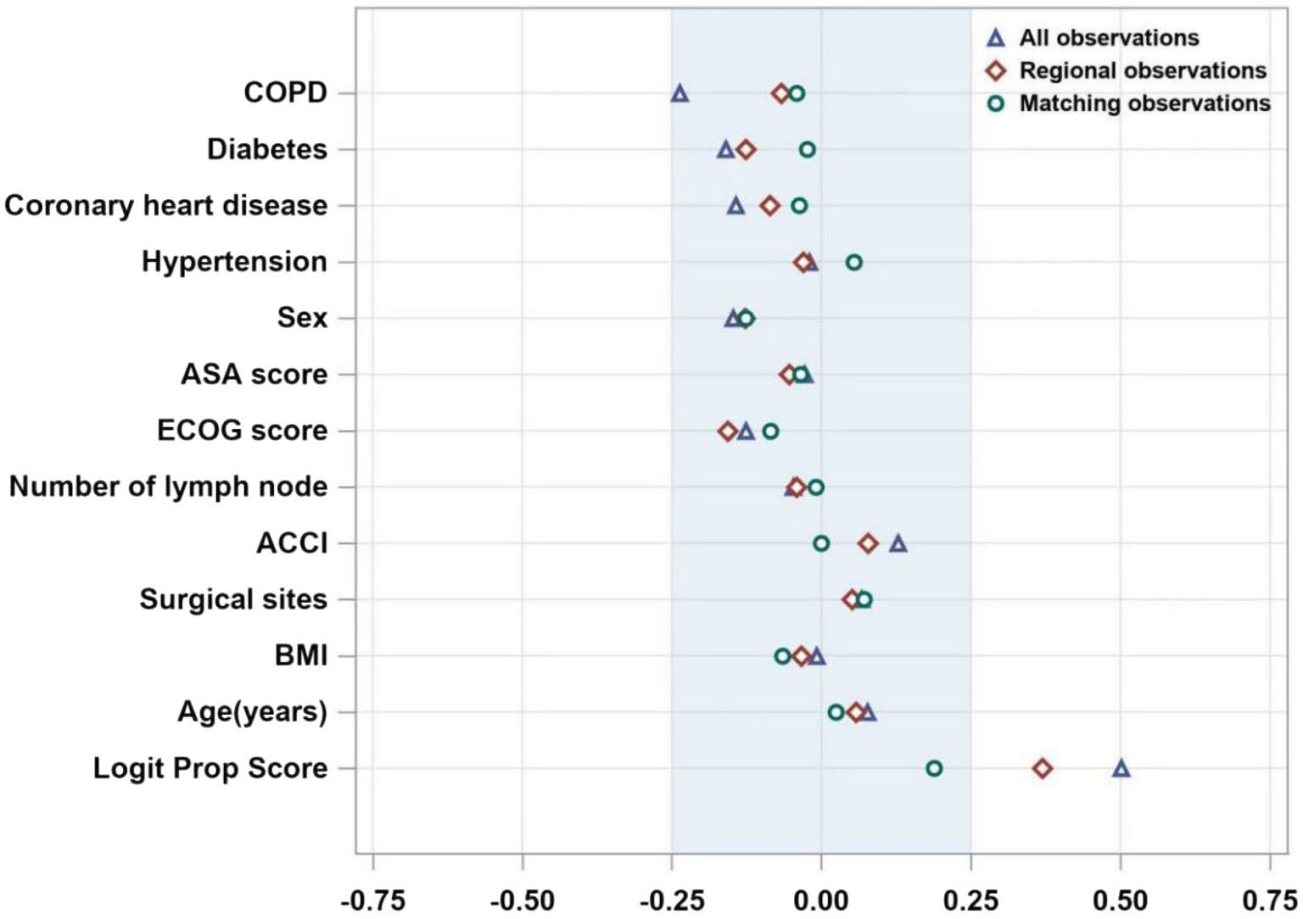
Propensity score matching. The two groups were matched for age, diabetes mellitus, coronary heart disease, COPD, hypertension, sex, ASA score, BMI, ECOG score, number of lymph node dissections, ACCI, and surgical site by caliper values (caliper values in the − 0.25 to 0.25 interval were included as matching factors) (Fig. 1)

Each independent variable was within the caliper value ranges (Fig. 1). Furthermore, as shown in Table 3, PSM produced two relatively balanced subgroups after the elimination of pre-matched COPD-related confounding effects.

**Table 3:** Comparison of the post-matching characteristics between the two groups [n (%)]

Variables	Warm-up group (n = 159)	Warm-up effect group (n = 159)	*χ* ^*2*^ */t*	*p-values*
**Sex**				
Female	90 (56.60)	80 (50.31)	1.26	0.261
Male	69 (43.40)	79 (49.69)		
**Age (years)**	59.7 ± 10.2	60.0 ± 9.7	0.21	0.835
**BMI**	22.3 ± 1.6	22.1 ± 1.7	0.58	0.559
**ACCI**	1.8 ± 1.1	1.8 ± 1.1	0.00	0.999
**Number of lymph node dissections**	5.2 ± 1.4	5.2 ± 1.3	0.08	0.934
**Hypertension**	22 (13.84)	19 (11.95)	0.25	0.616
**Diabetes mellitus**	9 (5.66)	10 (6.29)	0.06	0.813
**Coronary heart disease**	3 (1.89)	4 (2.52)	—	0.999*
**COPD**	0 (0.00)	1 (0.63)	—	0.999*
**Surgical anatomic site**			4.43	0.351
Right upper lobe	48 (30.19)	39 (24.53)		
Left upper lobe	31 (19.50)	43 (27.04)		
Right lower lobe	36 (22.64)	28 (17.61)		
Left lower lobe	31 (19.50)	32 (20.13)		
Right middle lobe	13 (8.18)	17 (10.69)		
**ECOG score**	0.7 ± 0.6	0.6 ± 0.7	0.84	0.402
**ASA score**	1.3 ± 0.5	1.3 ± 0.5	0.33	0.742

Results are expressed as propensity scores and *p-values*, where exceptions are stated separately. *p-values* > 0.05 indicated small differences

ASA: American Society of Anesthesiology score; BMI: body mass index; CAD: coronary artery disease; ACCI: age-adjusted Charlson Comorbidity Index; COPD: chronic obstructive pulmonary disease; ECOG score: Eastern Cooperative Oncology Group score

*Note*: *Fisher’s exact probability test; # Rank-sum test

### Comparison of primary outcomes and secondary outcomes between the two matched groups

The different primary outcomes of patients in the two matched groups are shown in Table 4.

**Table 4:** Comparison of primary outcomes between the two groups [n (%)]

Variables	Warm-up group (n = 159)	Warm-up effect group (n = 159)	*χ* ^*2*^ */t*	*p-values*
**Operation time (min)**	154.5 ± 54.9	147.2 ± 54.4	1.18	0.239
**Postoperative complications**	14(8.8)	22(13.8)	2.005	0.157
Pulmonary infection	3 (1.89)	1 (0.63)	—	0.623*
Pulmonary atelectasis	0 (0.00)	1 (0.63)	—	0.999*
Long-term pulmonary air leak	10 (6.29)	17 (10.69)	1.98	0.159
Poor incision healing	1 (0.63)	0 (0.00)	—	0.999*
Chylothorax	0 (0.00)	1 (0.63)	—	0.999*
Pleural effusion	0 (0.00)	2 (1.26)	—	0.498*

Results are expressed as propensity scores and *p-values*, where exceptions are stated separately. *p-values* > 0.05 indicated small differences

*Note*:*Fisher’s exact probability test; # Rank-sum test

The operation time did not significantly differ between the warm-up and warm-up effect groups (154.5 ± 54.9 vs. 147.2 ± 54.4 min, *p* = 0.239). Postoperative complications were graded based on the Clavien system (≥ grade 2). We noted 36 (11%) complications between warm-up and warm-up effect groups (14 [8.8] vs. 22 [13.8] cases, *p* = 0.157), including 4 (1%) cases of pulmonary infection, one (0.3%) case of pulmonary atelectasis, 27 (8.5%) cases of long-term pulmonary air leak, one (0.3%) case of poor incision healing, one (0.3%) case of chylothorax, and two (0.6%) cases of pleural effusion. Table 5 indicates the secondary outcomes between the two matched groups, including time to extubating of the chest drainage tube (4.6 ± 2.5 vs. 4.7 ± 3.2 days, *p* = 0.755), postoperative hospital stays (6.3 ± 3.0 vs. 6.2 ± 3.5 days, *p* = 0.890), and postoperative ICU admission (2 vs. 2 cases, *p* = 0.999).

**Table 5:** Comparison of secondary outcomes between the two groups [n (%)]

Variables	Warm-up group (n = 159)	Warm-up effect group (n = 159)	*χ* ^*2*^ */t*	*p-values*
Time to chest drainage tube extubation	4.6 ± 2.5	4.7 ± 3.2	0.31	0.755
Postoperative hospital stay	6.3 ± 3.0	6.2 ± 3.5	0.14	0.890
Postoperative ICU admission	2 (1.26)	2 (1.26)	—	0.999*

Results are expressed as propensity scores and *p-values*, where exceptions are stated separately. *p-values* > 0.05 indicated small differences

*Note*:*Fisher’s exact probability test; # Rank-sum test

## Discussion

Warm-up exercises, recognized for their skill-optimizing impact [15–18], were explored in this retrospective study for their potential influence on the prognosis of VATSL. However, the investigation revealed that patients in the operation warm-up effect group did not demonstrate quicker operating times or fewer postoperative complications compared to those in the operation warm-up group. No improvements in perioperative outcomes were observed. Therefore, when scheduling thoracic surgery, both doctors and patients may not need to consider the warm-up factor.

A prospective study by Maree K Weston [19], consistent with our results, failed to demonstrate the expected warm-up effect. Similarly, a retrospective study from McGill University in Canada found no significant improvement in perioperative results for epiretinal membrane stripping when surgical cases were warmed up [20], either through pre-surgery simulated warm-ups or actual surgical case warm-ups. However, these findings were limited by small sample sizes and variations in surgeon cases, thus lacking convincing evidence. To mitigate these limitations, our investigation utilized the largest sample size from the same surgeon in our center.

Additionally, the aforementioned studies focused on relatively straightforward procedures, and the sensitivity of warm-up effects might be more pronounced in more complex procedures. A larger sample size could yield unexpectedly diverse findings. A different retrospective study from the University of Pittsburgh compared 192 cases of laparoscopic sacroiliac pelvic fixation without warm-ups and 288 cases with warm-ups in terms of intraoperative complications, operation time, and postoperative hospital stay [21]. The operation durations in the two groups were 231.2 ± 55.2 vs. 225.9 ± 51.2 min, respectively. It was eventually noted that although the surgical warm-up did not affect the intraoperative complications of laparoscopic sacroiliac pelvic fixation, it marginally shortened the procedure duration and the length of time required for hospital recovery. Unfortunately, this study involved multiple surgeons without accounting for the impact of operation level or various doctors’ experience in large-scale surgery.

In our study, VATSL, requiring high proficiency and concentration, was selected to potentially highlight the impact of warm-up effects through a more challenging procedure. All surgeries were performed by the same thoracic surgeon; however, the results did not support the anticipated warm-up effect.

Throughout the study, the operator subjectively felt that after warming up the first operation, the second operation demonstrated improved skills in thoracoscopy instrument operation and internal thoracic organ anatomy. Nevertheless, what was not considered was that, after the first VATSL (operation time 154.5 ± 54.9 min), prolonged focused operation resulted in the operator accumulating a certain fatigue value, which may offset, to some extent, the proficiency improvement brought about by the warm-up effect. Therefore, considering relatively simple lung surgeries, such as lung wedge resection or simulator warm-up before proceeding with the first VATSL, may enhance the surgeon’s proficiency while mitigating the warm-up burden. Further research is warranted to validate these findings.

Warm-up not only improves surgical performance and short-term patient prognosis for novice surgeons but it can also assist experienced surgeons to complete a laparoscopic procedure faster [22]. Laparoscopy is a difficult and risky procedure, and should yield similar results. Kanav Kahol suggests that a warm-up prior to each surgery is valuable regardless of the experience of the surgeon. Participants of varying experience levels can benefit from warm-up. In a randomized study conducted by Thomas et al., warm-up and control groups of experienced and inexperienced surgeons were compared. They revealed that preprocedural warm-up improves task performance and reduces errors [23]. Cognitive arousal has been reported to cause increased somatic and cortical activity and can even assist with resisting sleep. The effects of short-term practice are not limited only to sports or physical activity. Thus, we believe that warm-up for surgeons is necessary regardless of their experience.

Our trial has several limitations. First, non-randomized selection bias was unavoidable because this was a single-center retrospective study. Second, the data in this study was procured from a single senior thoracic surgeon; therefore, it did not necessarily indicate the entire population. Lastly, due to the limited sample size, the warm-up (first) surgery and second surgery involved resection of the same lung lobe; thus, the warm-up effect could not be analyzed. In the future, we intend to conduct a multicenter prospective study on warming up while operating on the same lobe.

## Conclusion

In summary, regardless of whether the surgeon underwent warm-up, no significant distinctions were observed between the warm-up and warm-up effect groups concerning surgical time, early postoperative complications, thoracic duct removal time, or postoperative hospital stay. Consequently, the results of this study can offer guidance to thoracic surgeons and patients in making informed clinical decisions, particularly if concerns arise regarding the warm-up effect in surgery.

## Data Availability

The data underlying this article will be shared on reasonable request to the corresponding author.

## Abbreviations

VATSL: Video assisted thoracoscopic surgery lobectomy
BMI: Body mass index
ECOG: The Eastern Cooperative Oncology Group
ASA: American Society of Anesthesiology
ACCI: Age-corrected Charlson comorbidity index
COPD: Chronic obstructive pulmonary disease
